# TG2 as a novel breast cancer prognostic marker promotes cell proliferation and glycolysis by activating the MEK/ERK/LDH pathway

**DOI:** 10.1186/s12885-022-10364-2

**Published:** 2022-12-05

**Authors:** Dahai Xu, Ning Xu, Liang Sun, Zhaoying Yang, Miao He, Youjun Li

**Affiliations:** 1 Department of Human Anatomy, College of Basic Medical Sciences, Jilin University, 126 Xinmin Street, Changchun, Jilin 130021 P. R. China; 2 Department of Breast Surgery, China-Japan Union Hospital of Jilin University, Changchun, 130033 Jilin China; 3 Department of Anesthesia, The Second Hospital of Jilin University, 218 Ziqiang Street, Changchun, Jilin 130022 P. R. China

**Keywords:** Breast cancer, Prognosis, Tissue transglutaminase 2, Lactate dehydrogenase, Proliferation

## Abstract

**Background:**

Breast cancer (BC) is the most common malignant tumor among women worldwide. Tissue transglutaminase 2 (*TG2*) has been reported as a major player across several types of cancer. However, the effects of *TG2* in breast cancer are less known.

**Methods:**

The expression of *TG2* in patients with BC was detected by immunochemistry staining and RT-qPCR. The correlation of *TG2* expression and clinicopathological factors or overall survival (OS) was analyzed by Chi-square test, Kaplan-Meier, and Cox-regression analysis. The effects of *TG2* on cell proliferation and glycolysis were investigated in vivo and in vitro by gain- and loss-of-function experiments.

**Result:**

Both mRNA and protein levels of *TG2* were overexpressed in BC tissues and cultured cells. Clinical stage (*p* = 0.011), molecular subtype (*p*<0.001) and survival status (*p*<0.001) were significantly correlated with *TG2* expression. Specifically, *TG2* expression was positively associated with the clinical stage (r = 0.193, *p* = 0.005) and OS (r = 0.230, *p* = 0.001), while negatively associated with molecular subtype (r = − 0.161, *p* = 0.020). Overexpressed *TG2* was a prognostic factor of poor OS by Cox-regression analysis. Gain- and loss-of-function experiments indicated that cell proliferation and glycolysis were regulated by *TG2* via the MEK/ERK/LDH pathway. *TG2*-induced activation of the MEK/ERK/LDH pathway and glycolysis were attenuated by MEK inhibitor U0126.

**Conclusion:**

*TG2* is overexpressed in BC, which can serve as an independent prognostic factor for OS. *TG2* promotes tumor cell proliferation and increases glycolysis associated with the activation of the MEK/ERK/LHD pathway.

**Supplementary Information:**

The online version contains supplementary material available at 10.1186/s12885-022-10364-2.

## Introduction

Breast cancer (BC) is the most common malignant tumor among women worldwide and the second leading cause of cancer-related deaths [1]. Despite major advances in the diagnosis and treatment of BC in recent decades, the mechanism underlying tumorigenesis and progression is still unknown. Cancer is a multistep malignancy associated with mutation or abnormal expression of specific oncogenes and/or tumor suppressor genes [2]. The main cancer-promoting signaling pathways in BC are PI3K/AKT/mTOR and RAS/RAF/MEK, which control cell proliferation, invasion, and other biological functions [3, 4]. In addition, the mitogen-activated protein kinase (MEK) and extracellular signal-related protein kinase (ERK) pathway regulate lactate dehydrogenase (LDH) to reprogram cancer cell metabolism, which promotes glycolysis during cancer progression [5, 6]. Studies have revealed that while lactic acid promotes tumor metastasis, elevated LDH as a key enzyme in cancer metabolism acts as a poor prognostic biomarker [7, 8].

Tissue transglutaminase (TG) has been reported as a major player, across several types of cancer. TG can bind and hydrolyze GTP, as well as catalyze the enzymatic transamidation reaction which crosslinks primary amines to glutamine residues. TG adopts two vastly different conformations, depending on whether functioning as a GTP-binding protein or a crosslinking enzyme [9]. To date, nine human TGs have been identified since the discovery of the first TG (*TG2* or *TGM2*) by Heinrich Waelsch in 1957 [10]. Recent studies have shown that TG expression has relation to various biological functions which included extracellular matrix formation, apoptosis, cell migration, cell adhesion, and signal transduction [11]. Among the TGs, *TG2* is the most widely distributed and extensively studied. Several reports have supported increased expression of *TG2* in multiple cancer types, including BC, associated with poor disease outcome, increased drug resistance, and increased incidence of metastasis [12, 13]. These observations imply that *TG2* plays a crucial role in promoting an aggressive phenotype in mammary epithelial cells [14]. However, the underlying mechanism of *TG2* in tumors, especially in BC, is less studied.

In the present study, we found that both mRNA and protein levels of *TG2* are overexpressed in BC tissues and cultured cells, which also served as a poor prognostic factor of overall survival (OS). Gain- and loss-of-function experiments indicated that cell proliferation and glycolysis were regulated by *TG2* via the MEK/ERK/LDH pathway.

## Material and methods

### Cell lines and reagents

The human breast epithelium cell line MCF-10A was grown in DMED/HAM F12 1:1 mixed medium (Hyclone, Logan, UT, USA), which contains 5% horse serum (Gibco, Billings, MT, USA), hydrocortisone, cholera toxin, insulin (Sigma-Aldrich, St. Louis, MO, USA) and EFG (PeproTech, Cranbury, NJ, USA). The human BC cell lines SK-BR-3, BT-474 and MCF-7 were cultured in RPMI 1640, DMEM F12 or DMEM H21 (Hyclone), respectively, comprising 10% fetal bovine serum (Gibco). All cell lines were obtained from the American Type Culture Collection (Manassas, VA, USA) and cultured in a humidified atmosphere with 5-% CO_2_ at 37 °C. MEK inhibitor U0126 and DMSO was purchased from Sigma-Aldrich. A final concentration of 50 μM (1:1000, v/v) U0216 or the same volume of DMSO was applied to treat the cells.

### Patients and samples

The tissue samples of 210 female patients with BC who had received breast-conserving surgery or modified radical mastectomy during the period from January 2012 to December 2015 at China-Japan Union Hospital of Jilin University were enrolled in our study. The patients who received chemotherapy, radiotherapy, or endocrine therapy before surgery were excluded. The cases with recurrent or metastatic cancer at the time of diagnosis were also eliminated. The last date of follow-up was January 2020. Furthermore, a total of 40 normal or para-tumor tissues were acquired as controls. The study was approved by the Research Ethics Committee of the China-Japan Union Hospital of Jilin University and conducted with written informed consent from all patients.

### RT-qPCR

Total RNA extraction was isolated using TRIzol reagent (Ambion, Austin, TX, USA). The reverse transcription was performed with a 5 × PrimeScript RT Master Mix for Real-Time kit (TaKaRa, Tokyo, Japan). qRT-PCR was done using an SYBR® Premix DimerEraser™ Perfect Real-Time kit (TaKaRa). All operating procedures followed the supplier’s instructions. RT-qPCR analyses were done with a Mastercycler (Eppendorf, Hamburg, Germany). The primer sequences were as follow: TGM2, F: 5′- CAG CAG GGC TTT ATC TAC CA-3′, R: 5′- GAT CCC ATC TTC AAA CTG CC-3′; LDHA, F: 5′- TTC CAG TGT GCC TGT ATG G-3′ R: 5′- TTA TCA GTC CCT AAA TCT GGG TG-3′; LDHB, F: 5′- ACA ATA AGA TCA CTG TAG TGG G-3′, R: 5′- CAT CAG CCA GAG ACT TTC C-3′; GAPDH, F: 5′-GAA GGC TGG GGC TCA TTT GCA GGG-3′, R: 5′-GGT GCA GGA GGC ATT GCT GAT GAT-3′. The relative fold-change was normalized to GAPDH and calculated by the 2^-ΔΔCt^ method.

### Western blot

All the antibodies were obtained from Abcam (Cambridge, UK), except if stated otherwise. The cells were harvested in a lysis buffer and the proteins were extracted by RIPA buffer. The protein concentration was quantitated using the Bio-Rad (Hercules, CA, USA) method. Proteins (25 μg) were loaded into each well and separated on a 10% SDS-PAGE gel, and transferred to a nitrocellulose membrane. Blots were cut prior to hybridization with antibodies. After blocking with 5% fatty-free milk, the membranes were incubated with either *TG2*, p-MEK/MEK, p-ERK/ERK, LDHA/B, or GAPDH at a dilution of 1:1000 overnight. Anti-rabbit secondary antibodies were coupled to horseradish peroxidase (Proteintech, Wuhan, China). Immunoreactivity was detected using enhanced chemiluminescence (Beyotime, Shanghai, China) and visualized through autoradiography. Protein levels were normalized against GAPDH and analyzed.

### Immunochemistry staining (IHC)

To identify the level of *TG2* in BC tissues, IHC staining was performed. Briefly, 3 μm thick sections were deparaffinized, rehydrated and submerged into EDTA for antigen retrieval. All sections were then treated with hydrogen, heated, and incubated in bovine serum albumin. Subsequently, they were incubated with *TG2* antibody (Abcam) overnight at 4 °C. Negative controls were employed with normal goat serum. The sections were washed and incubated with secondary antibody and incubated with streptavidin-horseradish peroxidase complex (Invitrogen, Waltham, MA, USA). Each section was immersed in 3-amino-9-ethyl carbazole followed by counterstaining with Mayer’s hematoxylin, dehydrated, and then mounted.

The plasma staining of *TG2* was considered positive. The intensity of staining was categorized as follows: 0 (no staining), 1 (light yellow), 2 (yellow-brown), and 3 (brown). The percentage of positive cells were grouped as: 0% = 0, 1–10% = 1, 11–25% = 2, 25–50% = 3, and > 50% = 4. IHC intensity, with a range between 0 and 12, was calculated by the score of the staining indexes which equals the staining intensity × proportion of positive cells. The staining score of *TG2* level was grouped as: 0, no level; 1–3, weak; 4–6, mild; and > 6, strong.

### Establishment of *TG2* overexpression and shRNA knockdown cell colonies

Empty vector (EV), wildtype *TG2* for overexpression (OE), scramble control (SC), and shRNA targeting *TG2* (knockdown, KD) were constructed into lenti-virus (Hanbio, Shanghai, China). BT-474 cells were infected with wildtype *TG2* or EV. The cell line SK-BR-3 was infected with shRNA or SC. Concisely, 2 × 10^5^ cells were incubated with lenti-virus and polybrene (Hanbio) for 8 h. Fresh growth medium was added, and stable cell lines were gained in selected medium containing puromycin. Prepared cells were then used for further analysis as described.

### Cell proliferation assay

Cell proliferation was evaluated using a cell counting kit-8 (CCK-8, Meilunbio, Dalian, China). Cells were suspended into a 96-well plate in a 100 μL growth medium. After incubation for 24, 48, and 72 h, CCK-8 was added and further cultured for 4 h. The optical density (OD) value was obtained at 450 nm using a microplate reader (BioTek, Winooski, VT, USA).

### Glucose consumption and lactate production

The glucose/lactate assay kit (RSBio, Shanghai, China) was used to detect the levels of glucose and lactate in the cell culture medium following the manufacturer’s instructions. Briefly, 3 × 10^5^ cells/well were seeded into 6-well plates and incubated for 2 days. The medium was then replaced with a fresh medium and incubated for another 2 days. The medium was collected, and the glucose or lactate level was measured at the end of the incubation. The difference between the concentration of glucose or lactate and the controls (0 h) was used to calculate the glucose or lactate content. Glucose consumption, as well as lactate production, was normalized by cell numbers [15].

### Gene set enrichment analysis (GSEA)

The mRNA profile data were obtained from the TCGA database and included 1104 BC tissues and 114 normal tissues. The mRNA level of *TG2* was divided into a low- or high-level group based on the median ratio. Briefly, gene sets (h.all.v6.2.symbols.gmt) were obtained from the Molecular Signatures Database and analyzed using GSEA v3.0. Normalized enrichment scores (NES) were obtained from 1000 site permutations with a *p*-value < 0.05 and false discovery rate (FDR) < 0.25 considered statistically significant.

### Mice xenograft model

Six female nude mice (Huafukang Bioscience, Beijing, China) aged 4–5 weeks were randomly separated into two groups. SK-BR-3 cells (4 × 10^6^, either SC or KD) were injected into the fourth mammary fat pad of each mouse. The tumor size was measured using calipers every 3 days for 1 month. At the end of the experimental period, the mice xenograft tumors were excised and weighed after euthanasia. Organs such as the brain, heart, lung, and liver were fixed in formalin and pathologically investigated for signs of toxicity and metastatic lesions. All procedures of animal handling were approved by the Laboratory Animal Care and Use Committee of Jilin University.

### Statistical analysis

The statistical data were analyzed using SPSS v21.0 (IBM, Armonk, NY, USA). The Chi-square test as well as Spearman’ s correlation analysis was performed for analysis of the relationship between clinicopathological parameters and *TG2* expression. Survival curves drawn by the Kaplan-Meier method were compared using the log-rank test. Univariate and multivariate Cox-regressive analyses were performed for further assessment. Student’s *t*-test or one-way ANOVA was adopted to investigate data among or between groups. A *p*-value < 0.05 signified statistical significance. All the in vitro experimentations are performed independently at least three times, and the data were expressed as mean ± standard deviation.

## Results

### *TG2* overexpressed in patients with BC and an independent prognostic factor for OS

**Fig. 1:**
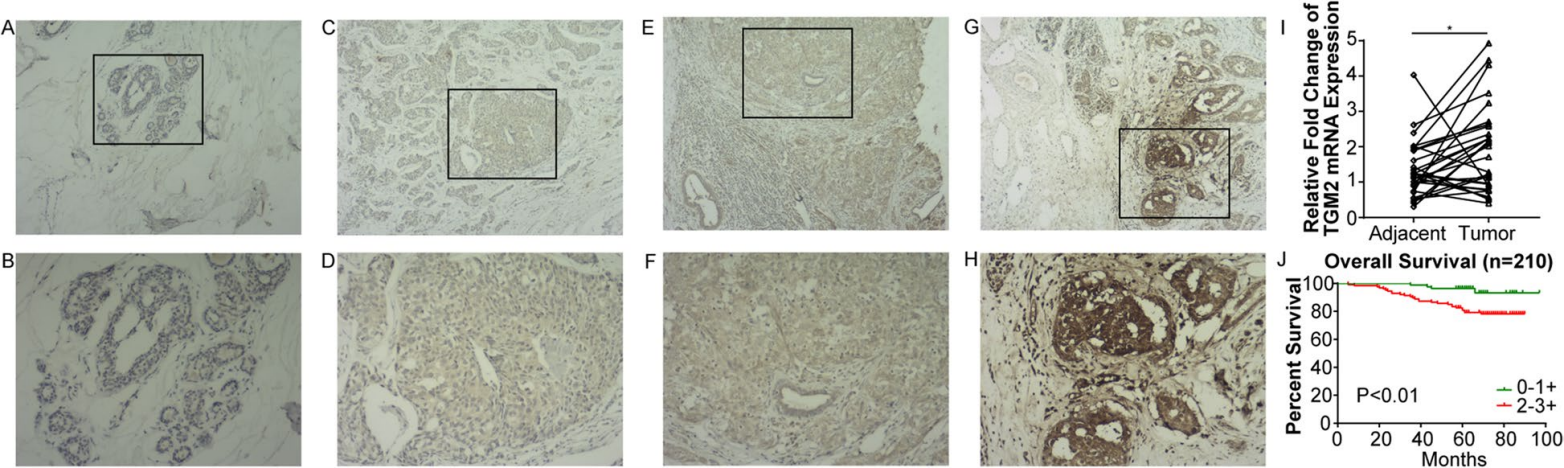
*TG2* is overexpressed in breast cancer tissues and correlated with poor survival. The representative images of immunochemistry staining of breast cancer tissues for negative (normal tissues, **A**, **B**), weak (1+, **C**, **D**), mild (2+, **E**, **F**) and strong (3+, **G**, **H**) *TG2* expression. The lower panel represents the magnification (× 200) of squared area from the upper panel (× 80). **I** the mRNA expression of *TG2* was performed in 40 paired adjacent and tumor samples by RT-qPCR. **J** High expression of *TG2* is correlated with poor overall survival in Kaplan-Meier analysis. (* *p*<0.05)

The present study enlisted 40 normal or adjacent samples and 210 BC samples. IHC staining showed negative or weak levels of *TG2* in normal or adjacent samples, while positive staining was observed in 183 out of 210 BC samples. According to the intensity of staining, the cohort was divided into two groups: negative to weak staining (0–1+) or mild to strong staining (2–3+) (Fig. 1A–H). In addition, higher levels of *TG2* mRNA were confirmed by RT-qPCR in 40 BC samples compared with the paired adjacent samples (Fig. 1I). Clinicopathological parameters were evaluated according to *TG2* expression using univariate analysis (Table 1). Clinical stage (*p* = 0.011), molecular subtype (*p*<0.001), and survival status (*p*<0.001) were significantly correlated with *TG2* expression. Next, Spearman correlation analysis was employed to estimate the correlation between *TG2* expression and clinicopathological characteristics in patients with BC (Table 2). *TG2* expression was positively associated with the clinical stage (r = 0.193, *p* = 0.005) and OS (r = 0.230, *p* = 0.001), while negatively associated with molecular subtype (r = − 0.161, *p* = 0.020). Kaplan-Meier analyses indicated that patients with high *TG2* expression represented significantly poorer OS (Fig. 1J). Furthermore, univariate and multivariate Cox-regression analysis demonstrated that high expression of *TG2* was a significant and independent prognostic factor for OS in patients with BC (Table 3).

**Table 1:** Correlation between clinicopathological characteristics and *TG2* expression in breast cancer samples

Characteristics	***TG2*** Expression		χ2	***P*** value
	0–1+	2–3+		
**Age (year)**				
<50	43	61		
≥ 50	41	65	0.156	0.693
**Menopause**				
No	47	65		
Yes	37	61	0.386	0.534
**MBNG**				
1	7	10		
2	57	94		
3	20	22	1.345	0.511
**Clinical stage**				
I	44	40		
II	25	52		
III	15	34	8.985	**0.011**
**T stage**				
1	56	76		
2	26	43		
3	2	6		
4	0	1	1.894	0.595
**N stage**				
0	50	64		
1	19	31		
2	9	17		
3	6	14	1.938	0.585
**ER**				
Negative	17	30		
Positive	67	96	0.370	0.543
**PR status**				
Negative	24	34		
Positive	60	92	0.064	0.801
**Her-2**				
Negative	41	64		
1+	19	20		
2+	19	29		
3+	5	13	2.399	0.494
**Ki-67**				
<30%	59	87		
≥ 30%	25	39	1.523	0.467
**Molecular subtype**				
Luminal A	21	51		
Luminal B (Her-2-)	12	33		
Luminal B (Her-2+)	32	14		
Her-2+	8	15		
Basal like	8	13	22.098	**<0.001**
**Survival**				
Yes	80	99		
No	4	27	17.864	**<0.001**

**Table 2:** Spearman correlation of *TG2* and clinicopathological characteristics

Characteristics	***TG2*** expression level	
	Spearman Correlation	***P*** Value
Clinical stage	0.193	0.005
Molecular subtype	−0.161	0.020
Survival	0.230	0.001

**Table 3:** Univariate and multivariate Cox-regression analysis of various prognostic parameters of overall survival in patients with breast cancer

Characteristics	Univariate analysis			Multivariate analysis		
	HR	95% CI	***P*** value	HR	95% CI	***P*** value
Clinical stage	2.268	1.416–3.634	0.001	2.106	1.298–3.418	0.003
Molecular subtype	1.225	0.955–1.570	0.110			
*TG2*	4.620	1.614–13.225	0.004	3.907	1.360–11.223	0.011

*HR* Hazard ratio, *CI* Confidence interval

### *TG2* promotes BC cell proliferation in vitro and in vivo

**Fig. 2:**
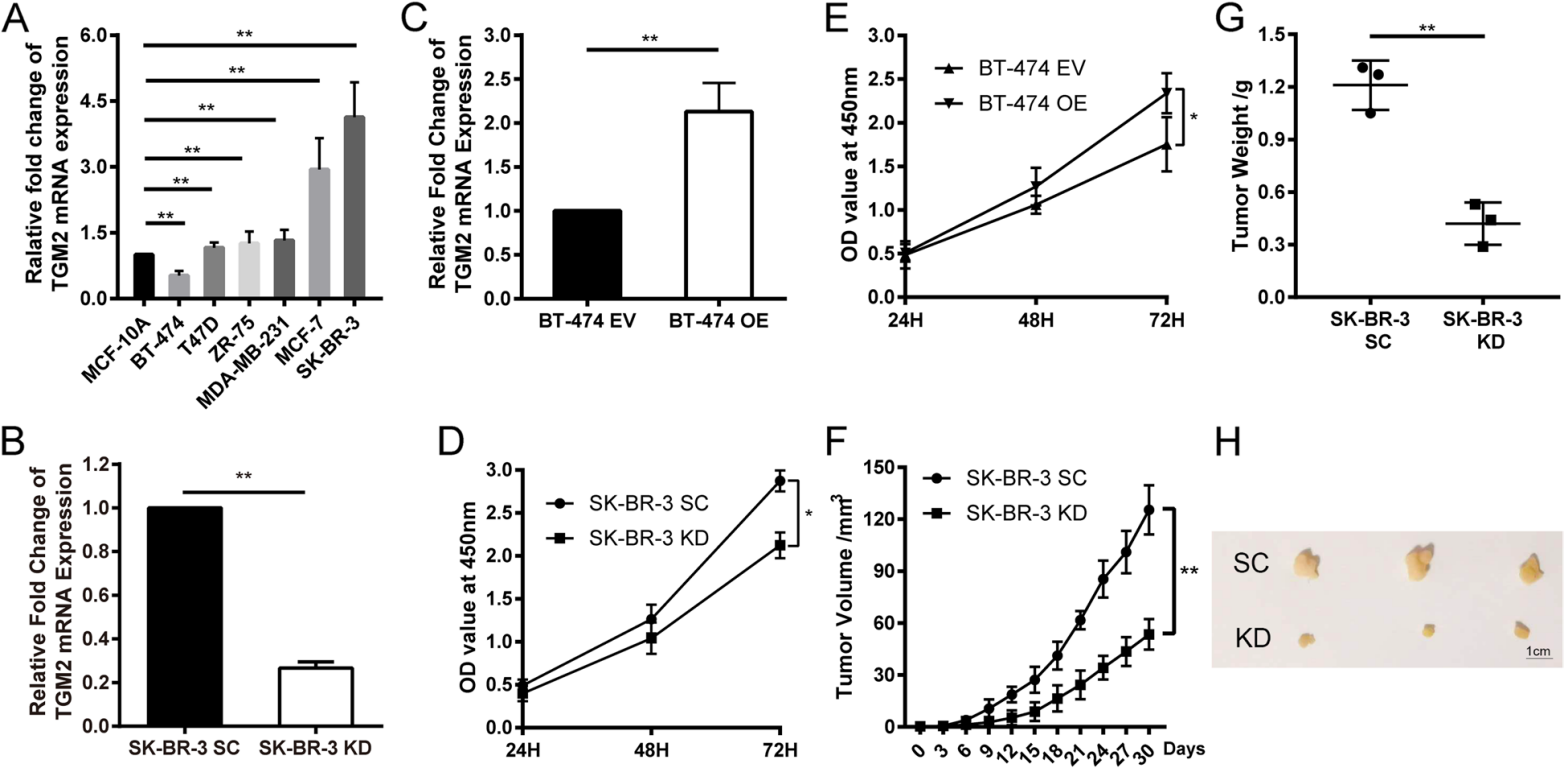
*TG2* is necessary for breast cancer proliferation in vivo and in vitro. **A** The mRNA expression of *TG2* was detected in breast cancer cell lines. **B** and **C** The *TG2* knockdown and overexpression cell line was established in SK-BR-3 and BT-474 cells, respectively. **D** and **E** The proliferation ability was measured by CCK-8 assay in *TG2* overexpression (BT-474) and knockdown (SK-BR-3) cell line. **F**-**H** The xenograft model was established in nude female mice by injection SK-BR-3 cells (scramble control  [SC] and knockdown [KD]), orthotopically. (* *p*<0.05, ** *p*<0.01)

**Fig. 3:**
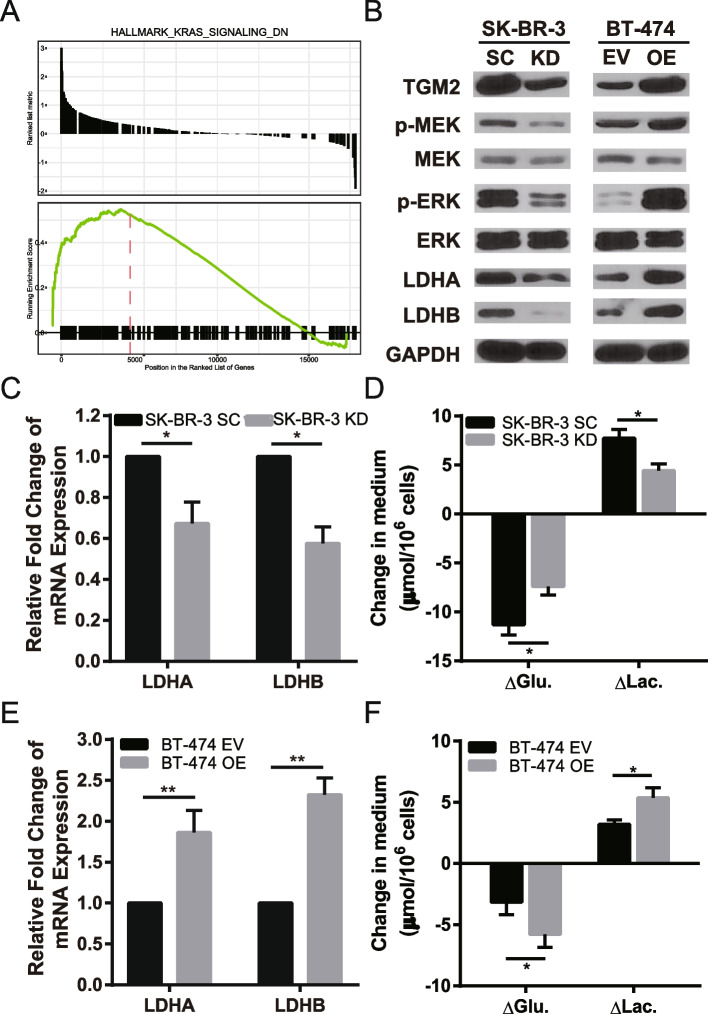
*TG2* regulated glycolysis via controlling MEK/ERK/LDH pathway. **A** GSEA analysis shown that KRAS pathway enriched. **B** p-MEK, p-ERK, LDHA and LDHB was down-regulated in *TG2* knockdown cell line (SK-BR-3), while upregulated in *TG2* overexpression cell line (BT-474) by western blot. **C** and **E** The mRNA level of LDHA and LDHB was detected by RT-qPCR in *TG2* knockdown and overexpression cell lines. **D** and **F** The consumption of glucose and production of lactate was measured in *TG2* knockdown and overexpression cell lines. (* *p*<0.05, ** *p*<0.01)

To understand the function of *TG2* in BC, the mRNA level of *TG2* was screened in several cell lines. Compared with normal breast epithelium MCF-10A cells, *TG2* expression was downregulated in BT-474, while higher in T47D, ZR-75, MDA-MB-231, MCF-7, and SK-BR-3 cells (Fig. 2A). Next, *TG2*-KD or OE cell lines were successfully generated in SK-BR-3 (mRNA level decreased by 73% in *TG2*-KD, *p* < 0.01) and BT-474 (mRNA level increased 2.13 times in OE, *p* < 0.01) cell lines, respectively (Fig. 2B–C, Fig. 3B). The cell proliferation ability was increased in *TG2*-OE (OD_450nm_ at 72 h, EV vs OE: 1.67 ± 0.12 vs 2.34 ± 0.13, *p* < 0.05) cell line (Fig. 2D), while inhibited in *TG2*-KD cell line (OD_450nm_ at 72 h, SC vs *TG2*-KD: 2.87 ± 0.07 vs 2.13 ± 0.09, *p* < 0.05) (Fig. 2E). Moreover, the xenograft model confirmed the inhibited growth in the *TG2*-KD cell line (Tumor weight: 1.21 ± 0.08 g vs 0.42 ± 0.07 g, tumor volume: 125.40 ± 8.26 mm^3^ vs 53.47 ± 5.10 mm^3^, *p* < 0.01) (Fig. 2F–H). Besides, no signs of toxicity and metastatic lesions were observed in the brain, heart, lung, liver, and other organs.

### MEK/ERK/LDH pathway and glycolysis were activated by *TG2*

To explore the potential mechanism by which *TG2* promotes BC cell proliferation, the bioinformatic analysis was used based on public database. GSEA showed that the KRAS pathway was enriched (Fig. 3A). Since MEK/ERK is the main downstream pathway of KRAS, and glycolysis is regulated by LDH via the ERK pathway [16], these key proteins were detected by western blot. Lower phosphorylation of MEK (58% decreased, *p* < 0.05) and ERK (37% decreased, *p* < 0.05) were observed in *TG2*-KD cells (Fig. 3B). Decreased protein and mRNA levels of LDHA (46% decreased, *p* < 0.05) and LHDB (31% decreased, *p* < 0.05) was also detected in these cells (Fig. 3B–C). The opposite phenomena were observed in the *TG2*-OE cell line, in which the higher phosphorylation of MEK (1.65-fold increased, *p* < 0.05) and ERK (1.78-fold increased, *p* < 0.05) resulted in increased levels of LDHA (2.77-fold increased, *p* < 0.01) and LDHB (1.62-fold increased, *p* < 0.01) (Fig. 3B and E). *TG2*-OE resulted in increased glycolysis (i.e., increased consumption of glucose and production of lactate by 2.63 ± 0.16 and 2.17 ± 0.87 μmol/10^6^ cells, *p* < 0.05), whereas *TG2*-KD decreased glycolysis (i.e., consumption of glucose and production of lactate decreased by 3.9 ± 1.04 and 3.3 ± 0.21 μmol/10^6^ cells, *p* < 0.05) (Fig. 3D and F, statistical significance for the changes in protein levels are shown in Supplementary Fig. 1). Taken together, these finding demonstrated the MEK/ERK/LDH axis and glycolysis activation by *TG2*.

### *TG2*-induced activation of MEK/ERK/LDH pathway and glycolysis attenuated by MEK inhibitor U0126

**Fig. 4:**
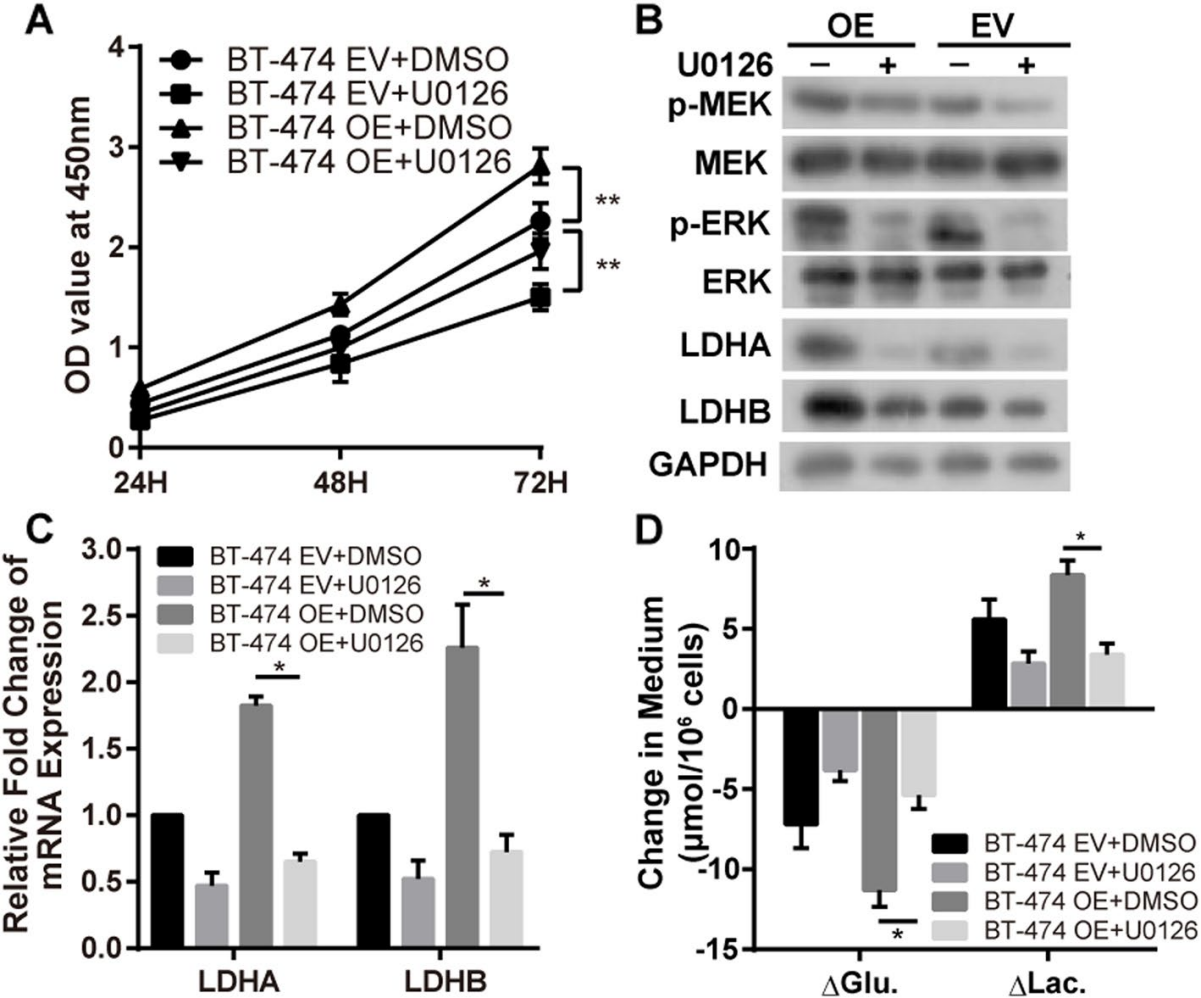
The proliferation and glycolysis promoted by *TG2* was attenuated by MEK inhibitor U0126. **A** and **D** The proliferation and glycolysis promoted by *TG2* was attenuated by U0126 treatment. **B** The activation of p-MEK, p-ERK, LDHA and LDHB by overexpressed *TG2* was rescued by U0126. **C** The mRNA level of LDHA and LDHB was detected by RT-qPCR. (* *p*<0.05, ** *p*<0.01)

To further confirm our hypothesis that *TG2* promotes BC cell proliferation by activation of the MEK/ERK/LDH pathway and reprogramming of cell metabolism, the MEK inhibitor U0126 was used to perform rescue experiments. The proliferation (OD_450nm_ of *TG2*-OE at 72 h, DMSO vs U0126: 2.74 ± 0.22 vs 1.93 ± 0.16, *p* < 0.01) and glycolysis (△Glu. and △Lac. of *TG2*-OE ± U0126: − 5.93 ± 0.17 and 4.97 ± 0.60 μmol/10^6^ cells, respectively, *p* < 0.01) promoted by *TG2* was attenuated by U0126 treatment (Fig. 4A and D). Furthermore, the activation of p-MEK, p-ERK, LDHA and LDHB by *TG2*-OE was rescued by U0126 (Fig. 4B and Supplementary Fig. 2). The same pattern of mRNA levels of LDHA (64% decreased, *p* < 0.01) and LDHB (68% decreased, p < 0.01) was detected by RT-qPCR (Fig. 4C).

## Discussion

Our study found that *TG2* was upregulated in both human BC tissues and cultured cells. A high mRNA level of *TG2* could serve as an independent prognostic factor of OS. *TG2*-OE promoted BC cell proliferation in vitro and in vivo. Moreover, glucose consumption and lactate production were shown to be regulated by *TG2*. All the findings indicated that cell proliferation and glycolysis were induced by *TG2* via activation of the MEK/ERK/LDH pathway. This hypothesis was further confirmed by rescue experiments in *TG2*-OE cells when treated with the MEK inhibitor U0126.

It is reported *TG2* is mainly a cytosolic protein but is present on the plasma membrane, in the nucleus, and the extracellular environment [11]. Our findings are consistent with previous reports showing elevated *TG2* expression in ovarian cancer [17], lung cancer [18], and glioblastoma [19]. Numerous studies indicated that elevated expression of *TG2* in cancer tissues is related to poor survival, drug resistance, and metastasis [1]. For instance, Jiang et al. reported that normal breast tissues expressed low levels of *TG2*, while node-positive tumors exhibited significantly higher levels of *TG2* [20]. Mehta et al. found up-regulated expression of *TG2* in MDA-MB-231 cells and the metastatic lymph nodes of patients with BC [21]. Hettasch et al. reported up-regulated expression of *TGs* in intraductal and invasive BC, which its localization in the neovasculature and extracellular matrix suggested that TGs may be involved in the regulation of tumor growth and metastasis [22]. Furthermore, Kyparidou et al. reported that TG is an independent favorable prognostic factor for OS in a cohort of 68 patients with BC [23]. Although *TG2* is extensively studied in many types of cancer, its prognostic role is controversial in BC and the mechanism by which it promotes the progression of BC is less explored. Our data indicated that *TG2* may be a novel biomarker for BC.

To further explore the underlying mechanism of *TG2* promotion of BC cell proliferation, we found that *TG2*-OE or -KD respectively elevated or reduced phosphorylation of MEK/ERK. These findings revealed that *TG2* may promote BC proliferation by activating the MEK/ERK pathway. Rapidly growing tumor cells acquire genetic and epigenetic alterations to meet their oxygen requirements via upregulation of glycolysis. Once the cells are exposed to various stress stimuli, mechanisms such as metabolic reprogramming and autophagy will be induced to provide nutrients and metabolites to the stressed cells [24, 25]. A continuous supply of energy is required to produce precursors for de novo synthesis of biomacromolecules involved in cell growth and proliferation, including RNA, DNA, amino acids, fatty acids. This energy can be provided by the reprogramming of glucose metabolism. For instance, hypoxia-inducible factor-1 (HIF-1) is a transcription factor that can create a shift in energy production from mitochondria towards glycolytic sources in tumor hypoxia regions, and its upregulation forms the main mechanism of metabolic reprogramming [26]. *TG2* reportedly increases the Warburg effect by inducing HIF-1α via NFκB in BC cells, resulting in increased glucose uptake, which could be abolished by KD of HIF-1α, NFκB, or *TG2* [27–29]. Furthermore, glycolysis in tumor cells is reportedly regulated by LDH through the MEK/ERK pathway [6]. Accordingly, respective higher or lower expression of *LDH* was also seen in *TG2*-OE or -KD cells in the present study. This result implicates the involvement of the *TG2*/MEK/ERK/LDH axis during BC progression.

LDH, with two major subunits LDHA and LDHB, can catalyze a mutual reversible conversion between pyruvic acid and lactic acid. Research has shown that lactate acid, an end product of glycolysis, can versatilely promote tumor growth and participates in the metabolic symbiosis of tumors [30]. Recent studies have shown that the mitochondrial oxidative phosphorylation pathway is downregulated, while glucose consumption and lactate release rate increased in cancer cells, independent of oxygen availability (Warburg effect) [31]. Furthermore, anaerobic glycolysis produces large amounts of lactate, which is a primary substrate for the TCA cycle in cancer cells [32]. It is widely accepted that this metabolic reprogramming promotes the growth and survival of tumors. LDH is a key enzyme involved in cancer metabolism could serve as a prognostic index, as the elevated level is linked to poor prognosis [7, 33]. Previous research on reduced LDH expression has also shown that LDH participated in tumorigenesis, however, the relevant mechanism has not been elucidated [34]. Many molecules, such as MYC, KRAS, and the tumor suppressor TP53, are involved in the regulation of metabolic glycolysis as well as oxidative stress, although their underlying molecular mechanisms also remain unclear [35]. LDH may be an emerging target for cancer therapy in pharmacological approaches among the enzymes involved in glycolysis [36].

Moreover, the presence of *TG2* in the extracellular environment is crucial for tumor progression. It is reported that high levels of *TG2* and fibrillar fibronectin detected in BC-derived extracellular vesicle promotes BC cell growth in a *TG2*-dependent model [12]. In addition, fibronectin could promote BC cell metastasis as well [37, 38], while autocrine fibronectin inhibits BC metastasis [39]. It is reported that *TG2* forms a complex with fibronectin to play a role in fibronectin-mediated BC cell attachment, growth, and survival functions [40]. However, the interaction between *TG2* and fibronectin needs further investigation. Considering the complication of crosstalk between the main signaling pathways and their feedback loops, future research also needs to investigate the interaction between *TG2* with RAS/RAF or other pathways.

Another limitation of our study is that the cell lines used for gain- or loss-of-function were based on the level of *TG2*. BT-474 was isolated from primary tumors which is an HR^−^ and Her-2^+^ subtype, while SK-BR-3 was isolated from metastatic tissue, which is an HR^+^ and Her-2^+^ subtype. As these receptors may be important in the activation of the MEK/ERK/LDH pathway, further study is needed. It is reported that EGFR is mediated by TG2 phosphorylation and suggested the targeting TG2 as a novel strategy to downregulate EGFR signaling [41]. According to these findings, our further plan is exploring the effects of TG2 on HER2 or EGFR, and the sensitivity of tyrosine kinase inhibitors or HER2 target reagents such as trastuzumab etc.

Noticeable, our results showed that MEK inhibitor U0126 decreased p-MEK while MEK is phosphorylated by RAF kinase (Fig. 4B). Xuening et al. reported that phosphorylation of RAF-1 can be directly blocked by compounds specific inhibitors of MEK activation [42]. Kinase suppressor of Ras (KSR-1), a kinase upstream of Raf-1, is a substrate of ERK. U0126 as well as PD 098059 inhibits the phosphorylation activity of KSR-1 at concentrations similar to those that inhibit MEK-1. In addition to its target RAF-1, KSR-1 has been reported to associate with ERK and MEK proteins. Moreover, Eppstein et al. explored MAPK signaling and growth response in three neuroblastoma cell types after U0126 inhibition [43]. p-ERK levels decreased in dose response to U0126 at 1 and 24 hours in all lines. Conversely, p-MEK levels increased with increasing U0126 concentrations at 24 hours in all lines. The shRNA targeting MEK1/2 may be applied for further investigation. In addition, the effect of U0126 on KSR-1 and the association of KSR-1 with MEK/ERK or TG2 in breast cell lines also need more research.

In conclusion, our present study showed that *TG2* is overexpressed in BC and acts as an independent prognostic factor for OS. Gain- and loss-of-function experiments indicated that *TG2* promotes tumor cell proliferation and increases glycolysis by activating the MEK/ERK/LHD pathway.

## Supplementary Information

**Additional file 1: Supplementary fig. 1.** Densitometric analysis for western bolt: A. TG2 knockdown in SK-BR-3; B. TG2 overexpress in BT-474. (*: *p* < 0.05). **Supplementary fig. 2.** Densitometric analysis for western bolt of U0126 treatment in TG2 overexpressed SK-BR-3 cells.

**Additional file 2.**

**Additional file 3.**

**Additional file 4.**

**Additional file 5.**

## Data Availability

All data generated or analyzed during this study are included in this published article.

## Abbreviations

BC: Breast cancer
CCK-8: Cell counting kit-8
ERK: Extracellular signal-related protein kinase
EV: Empty vector
FDR: False discovery rate
GSEA: Gene set enrichment analysis
HIF-1: Hypoxia-inducible factor-1
IHC: Immunochemistry staining
KD: Knockdown
LDH: Lactate dehydrogenase
MEK: Mitogen-activated protein kinase kinase
NES: Normalized enrichment scores
OD: Optical density
OE: Overexpression
OS: Overall survival
SC: Scramble control
TG: Tissue transglutaminase
