# Supplementary material for: TG2 as a novel breast cancer prognostic marker promotes cell proliferation and glycolysis by activating the MEK/ERK/LDH pathway
Source: BMC Cancer. 2022 Dec 5;22:1267. doi: 10.1186/s12885-022-10364-2 (PMC9724448; doi:10.1186/s12885-022-10364-2)
Supplement: Supplementary file 1 — Additional file 1: Supplementary fig. 1. Densitometric analysis for western bolt: A. TG2 knockdown in SK-BR-3; B. TG2 overexpress in BT-474. (*: p < 0.05). Supplementary fig. 2. Densitometric analysis for western bolt of U0126 treatment in TG2 overexpressed SK-BR-3 cells. [file 12885_2022_10364_MOESM1_ESM.docx]

Supplementary figure 1. Densitometric analysis for western bolt: A. TG2 knockdown in SK-BR-3; B. TG2 overexpress in BT-474. (*: p<0.05)


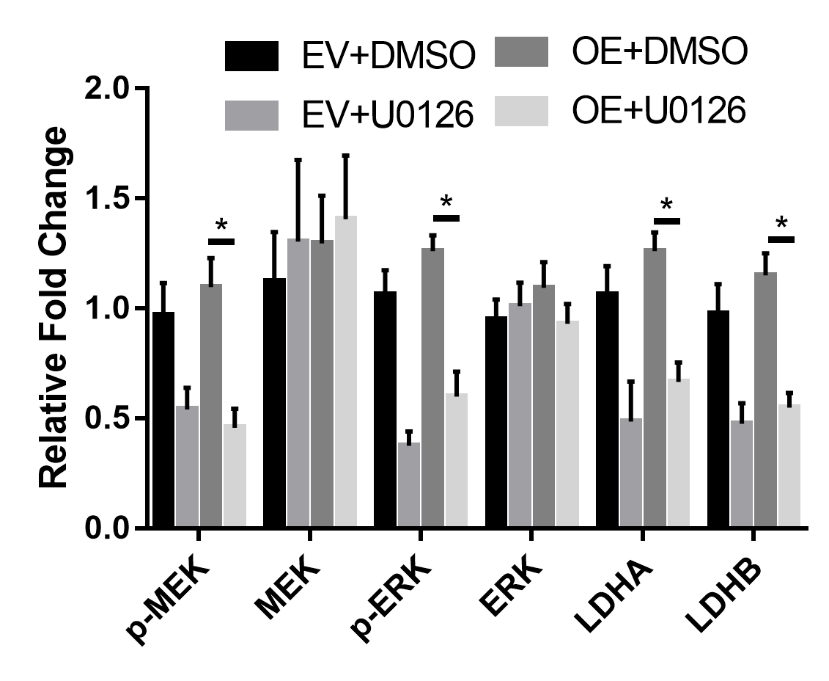


Supplementary figure 2. Densitometric analysis for western bolt of U0126 treatment in TG2 overexpressed SK-BR-3 cells.
