# Supplementary figures and images for: TG2 as a novel breast cancer prognostic marker promotes cell proliferation and glycolysis by activating the MEK/ERK/LDH pathway
Source: BMC Cancer. 2022 Dec 5;22:1267. doi: 10.1186/s12885-022-10364-2 (PMC9724448; doi:10.1186/s12885-022-10364-2)

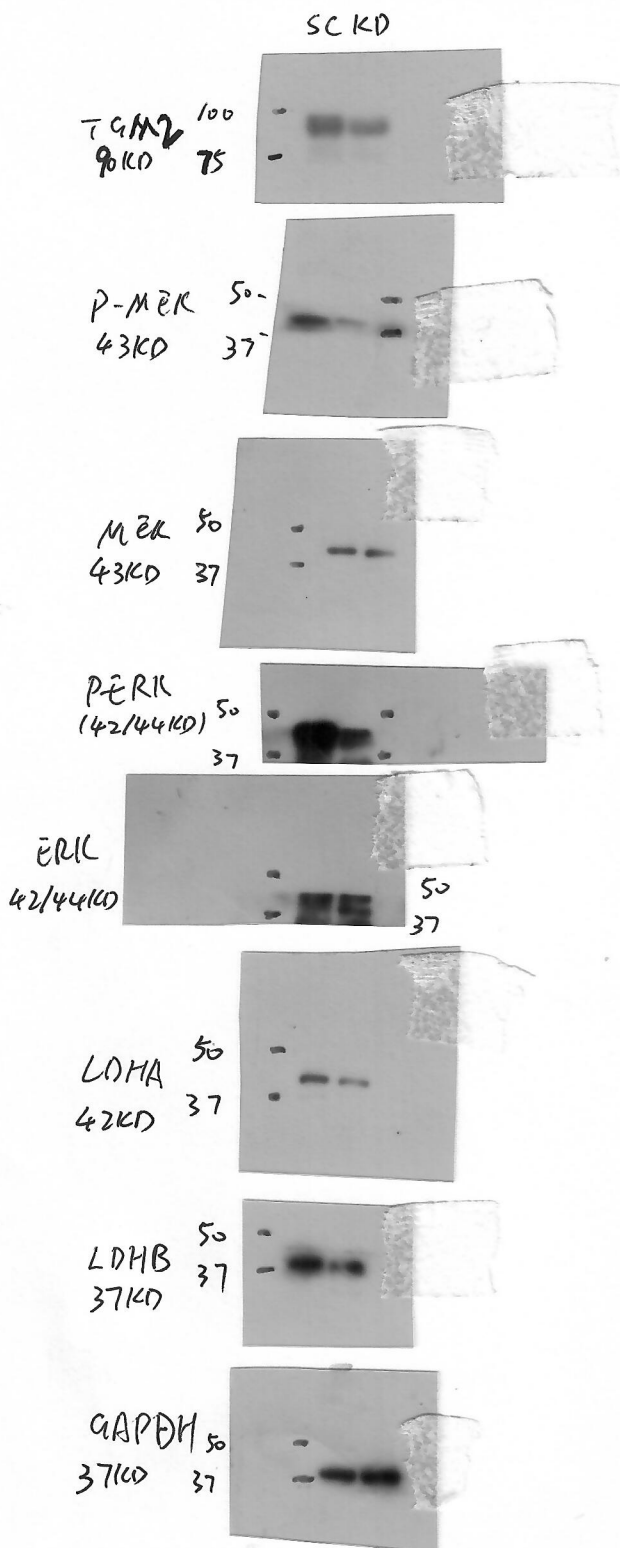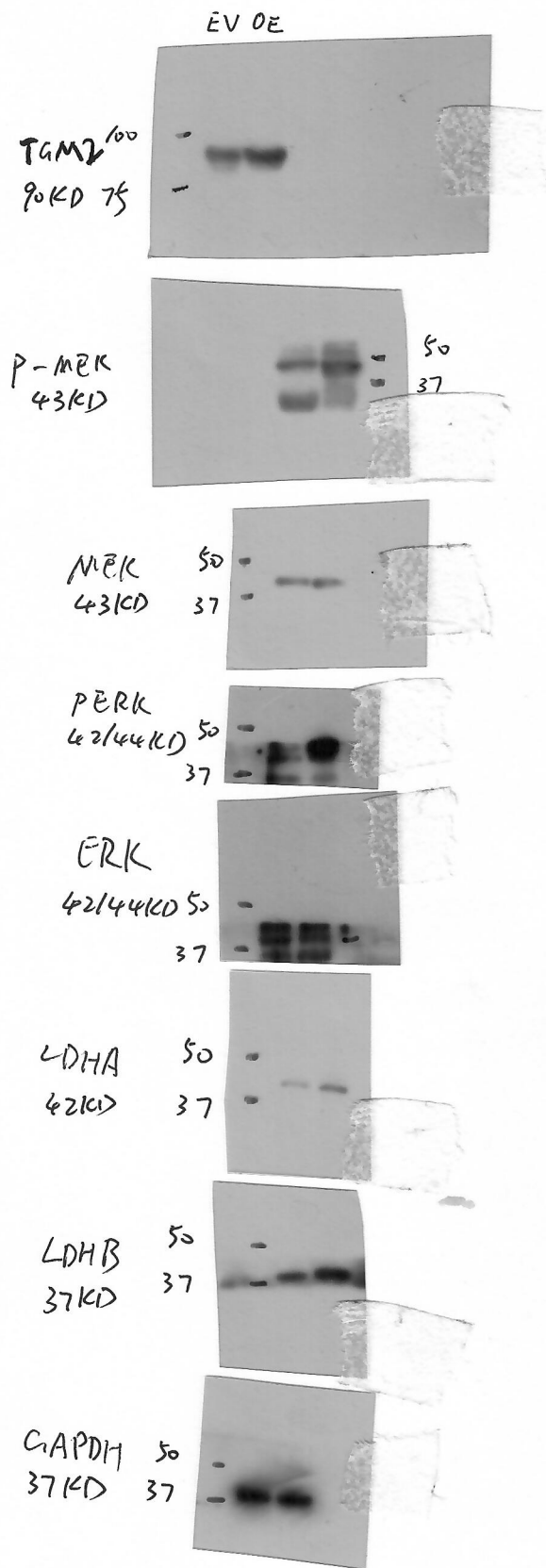

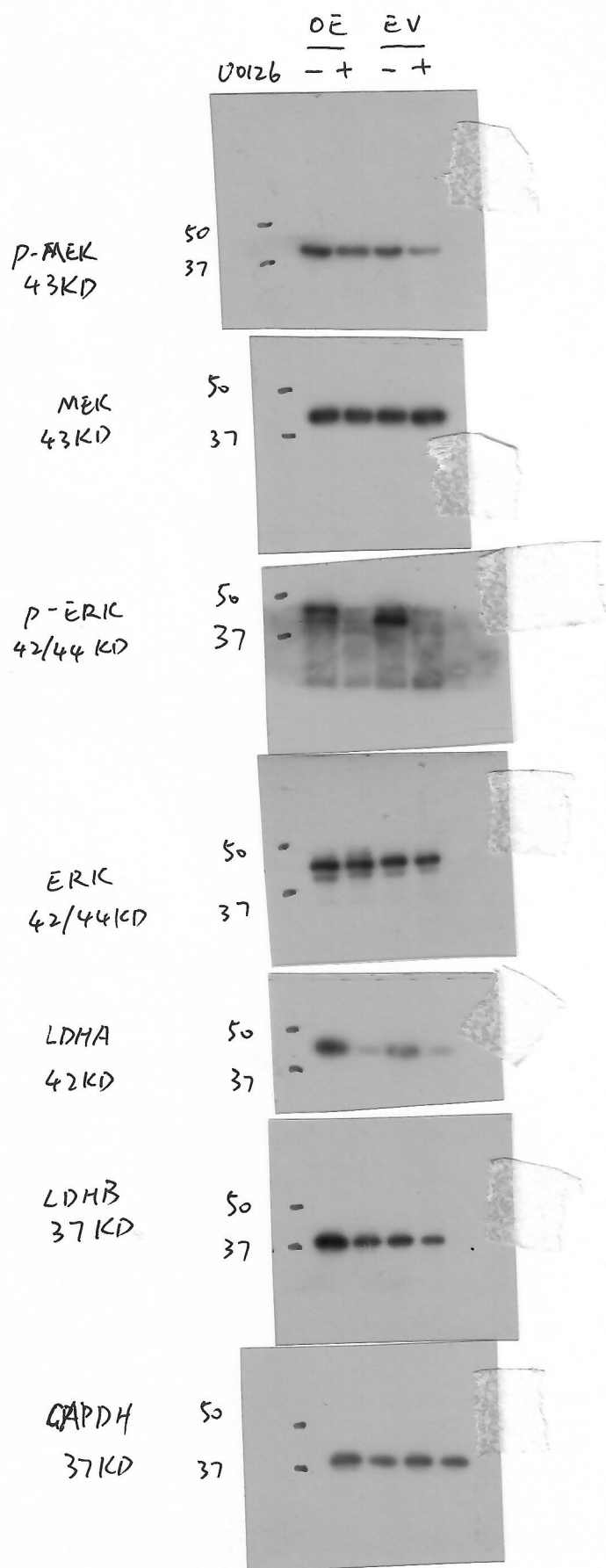

Supplement: Supplementary file 2 — Additional file 2. [file 12885_2022_10364_MOESM2_ESM.pdf]
