# Supplementary material for: TG2 as a novel breast cancer prognostic marker promotes cell proliferation and glycolysis by activating the MEK/ERK/LDH pathway
Source: BMC Cancer. 2022 Dec 5;22:1267. doi: 10.1186/s12885-022-10364-2 (PMC9724448; doi:10.1186/s12885-022-10364-2)
Supplement: Supplementary file 3 — Additional file 3. [file 12885_2022_10364_MOESM3_ESM.pdf]

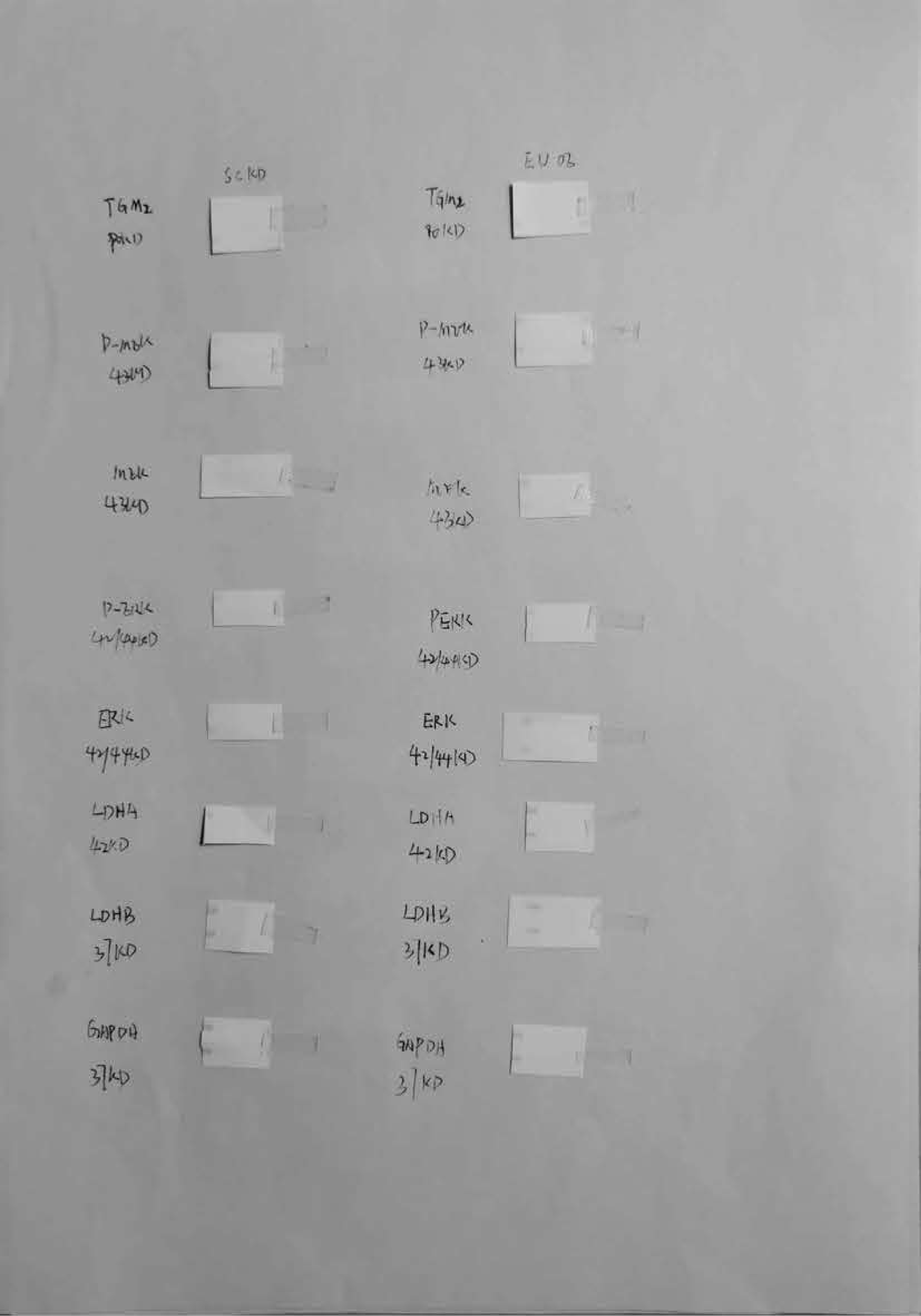
SCKD
EV OB
TGM2 90KD
P-MEK 43KD
MEK 43KD
P-ERK 42/44KD
ERK 42/44KD
LDHA 42KD
LDHB 37KD
GAPDH 37KD
TGM2 90KD
P-MEK 43KD
MEK 43KD
PERK 42/44KD
ERK 42/44KD
LDHA 42KD
LDHB 37KD
GAPDH 37KD

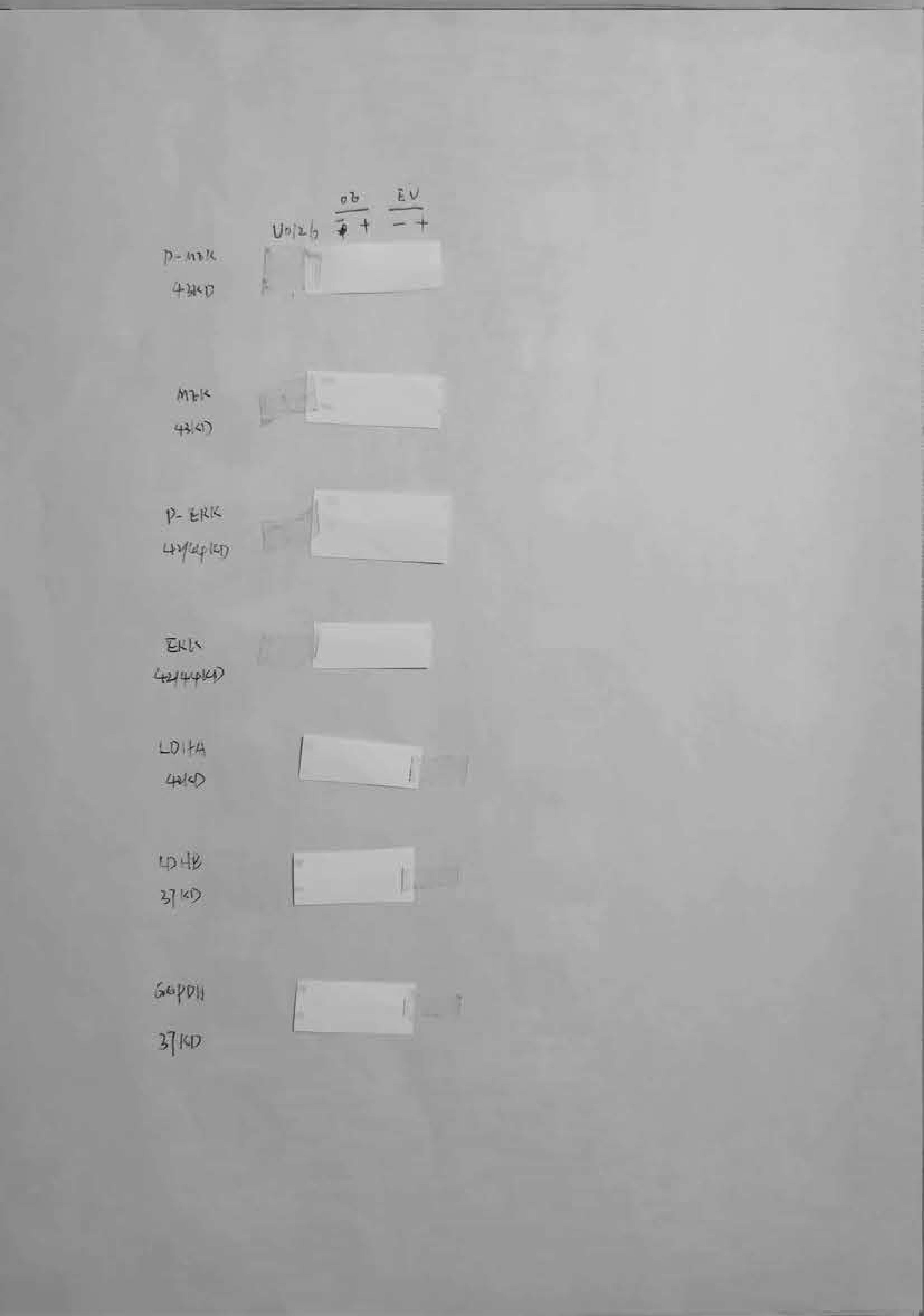
OB
EV
U0126
- +
- +
P-MEK 43KD
MEK 43KD
P-ERK 42/44KD
ERK 42/44KD
LDHA 42KD
LDHB 37KD
GAPDH 37KD
