# Supplementary material for: TG2 as a novel breast cancer prognostic marker promotes cell proliferation and glycolysis by activating the MEK/ERK/LDH pathway
Source: BMC Cancer. 2022 Dec 5;22:1267. doi: 10.1186/s12885-022-10364-2 (PMC9724448; doi:10.1186/s12885-022-10364-2)
Supplement: Supplementary file 4 — Additional file 4. [file 12885_2022_10364_MOESM4_ESM.pdf]

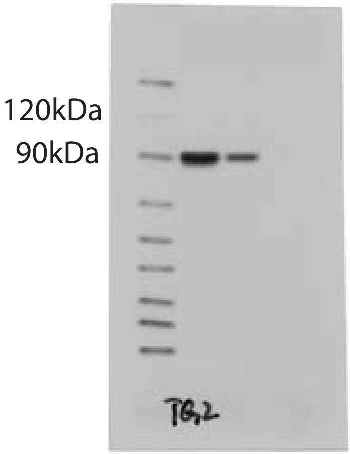

2nd-Fig3-SKBR3-SC and KD

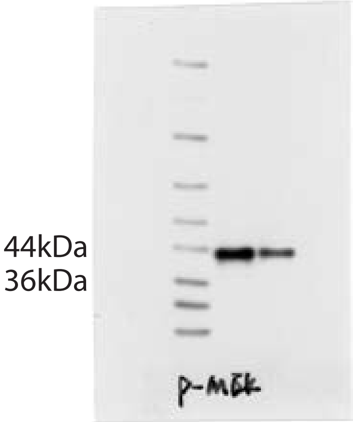

2nd-Fig3-SKBR3-SC and KD

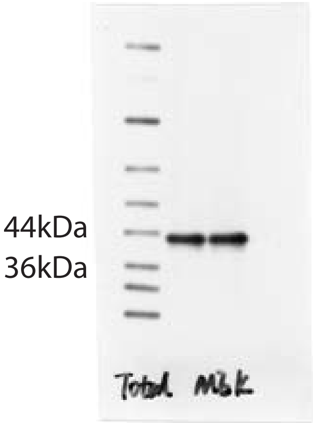

2nd-Fig3-SKBR3-SC and KD

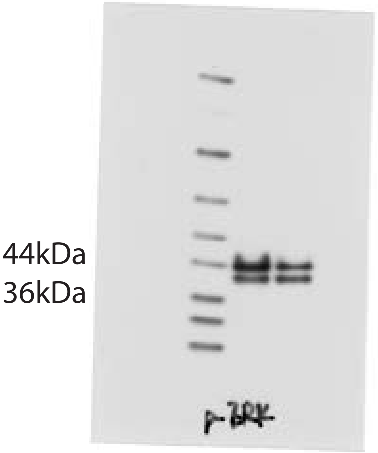

2nd-Fig3-SKBR3-SC and KD

44kDa
36kDa

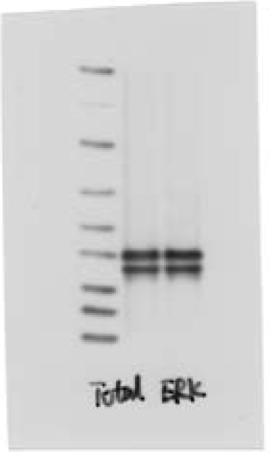

2nd-Fig3-SKBR3-SC and KD

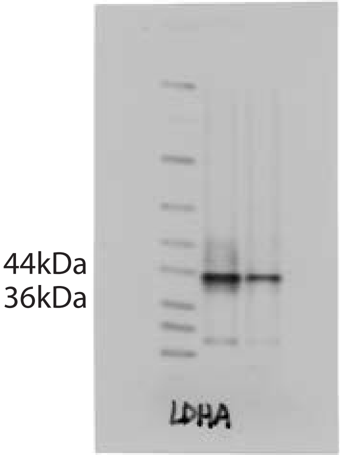

2nd-Fig3-SKBR3-SC and KD

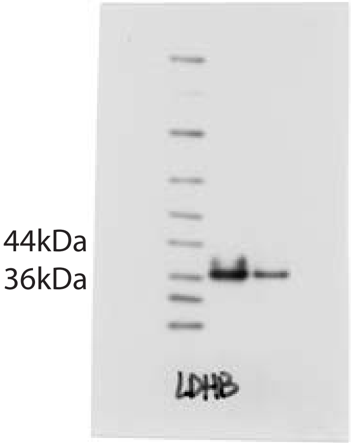

2nd-Fig3-SKBR3-SC and KD

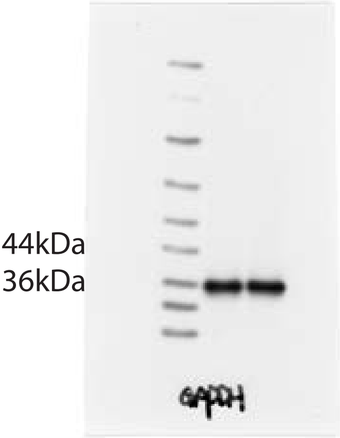

2nd-Fig3-SKBR3-SC and KD

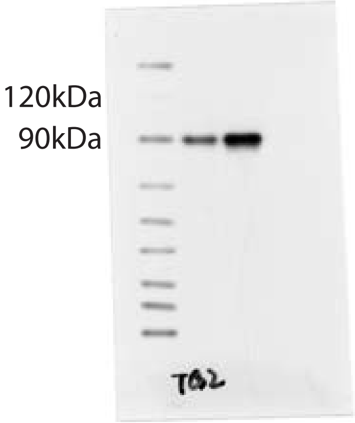

2nd-Fig3-BT474-EV and OE

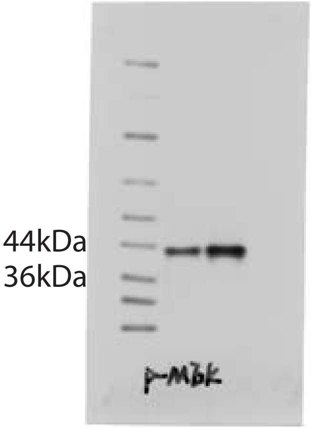

2nd-Fig3-BT474-EV and OE

44kDa
36kDa

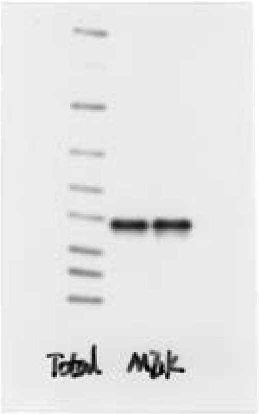

2nd-Fig3-BT474-EV and OE

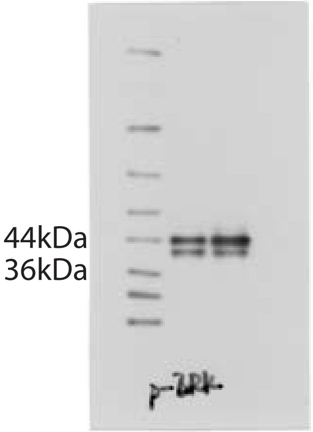

2nd-Fig3-BT474-EV and OE

44kDa
36kDa

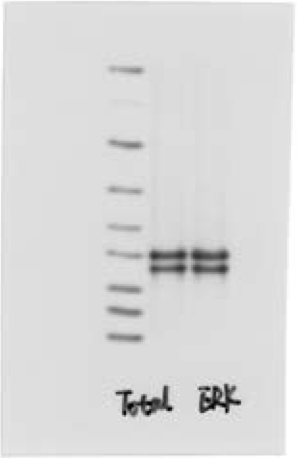

2nd-Fig3-BT474-EV and OE

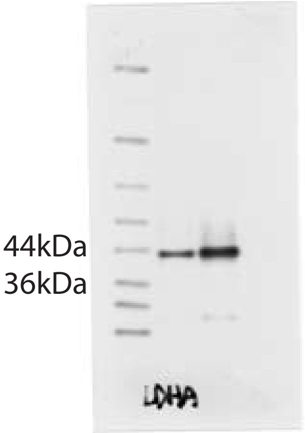

2nd-Fig3-BT474-EV and OE

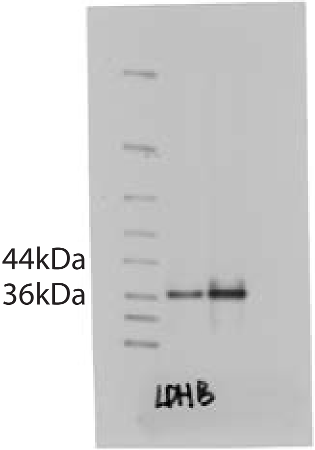

2nd-Fig3-BT474-EV and OE

44kDa
36kDa

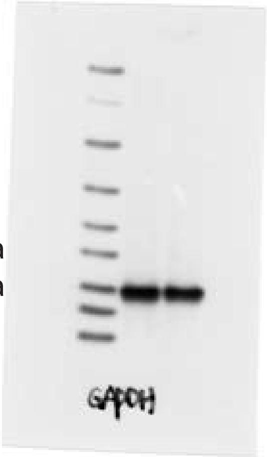

2nd-Fig3-BT474-EV and OE

44kDa
36kDa

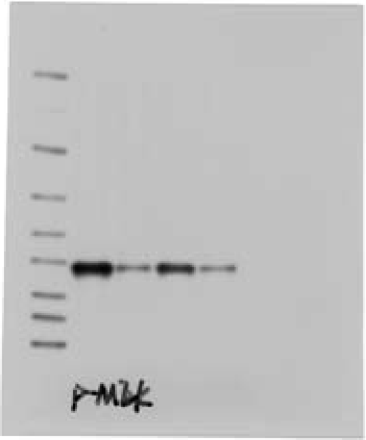

2nd-Fig4

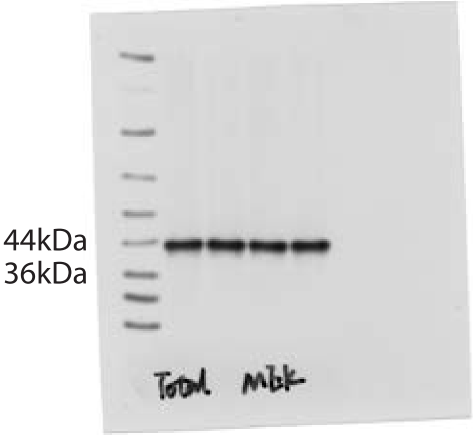

2nd-Fig4

44kDa
36kDa

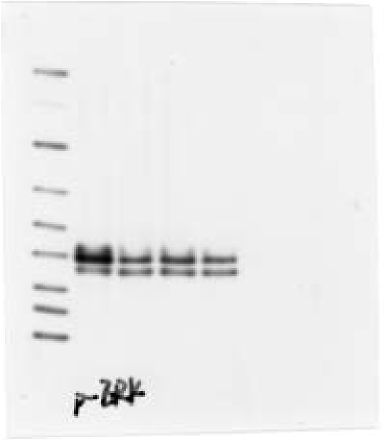

2nd-Fig4

44kDa
36kDa

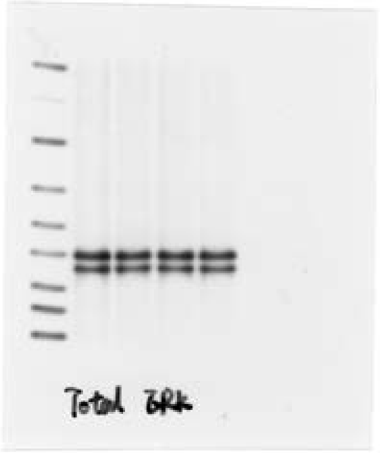

2nd-Fig4

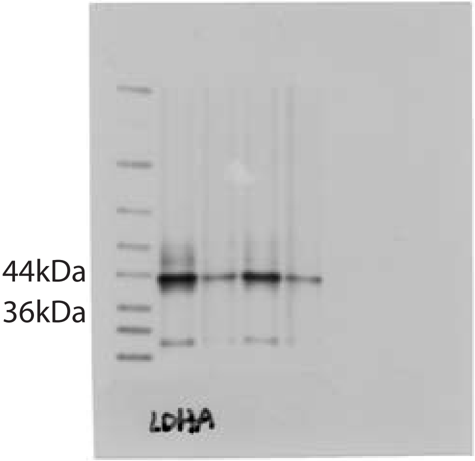

2nd-Fig4

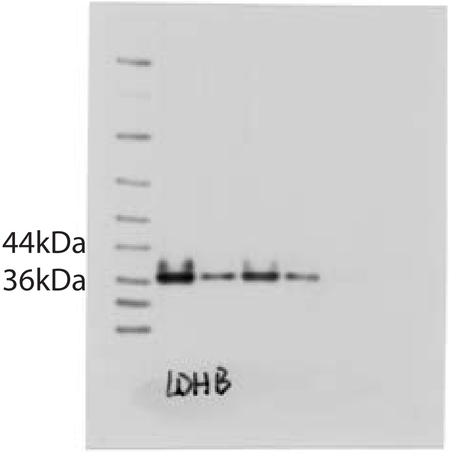

2nd-Fig4

44kDa
36kDa

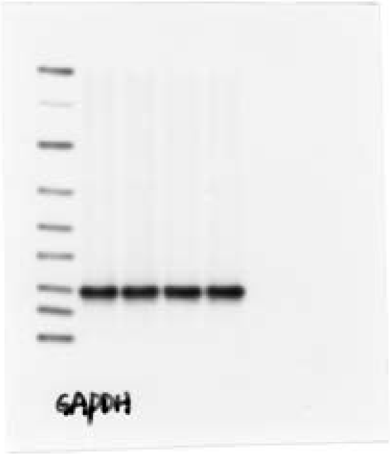

2nd-Fig4
